# Modulation of Charge Transport at Grain Boundaries in SrTiO_3_: Toward a High Thermoelectric Power Factor at Room Temperature

**DOI:** 10.1021/acsami.0c21699

**Published:** 2021-03-04

**Authors:** Jianyun Cao, Dursun Ekren, Yudong Peng, Feridoon Azough, Ian A. Kinloch, Robert Freer

**Affiliations:** † Department of Materials, University of Manchester, Oxford Road, Manchester M13 9PL, U.K.; ‡ National Graphene Institute and Henry Royce Institute, University of Manchester, Oxford Road, Manchester M13 9PL, U.K.; § Department of Metallurgy and Materials Engineering, Iskenderun Technical University, Iskenderun 31200, Hatay, Turkey

**Keywords:** SrTiO_3_, thermoelectric, grain boundary, modulation, charge transport

## Abstract

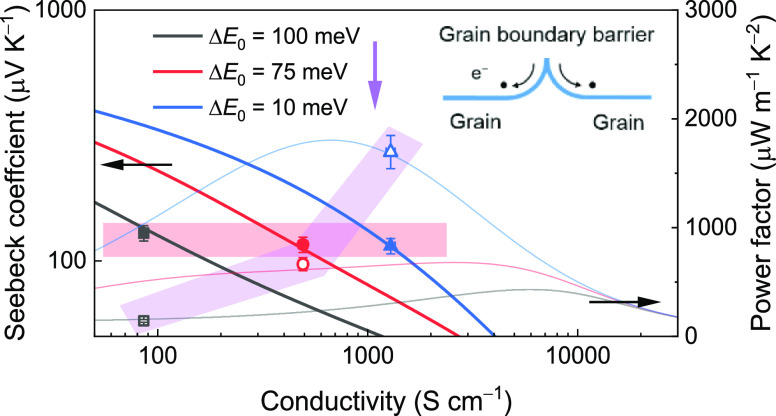

Modulation of the grain boundary properties in thermoelectric materials that have thermally activated electrical conductivity is crucial in order to achieve high performance at low temperatures. In this work, we show directly that the modulation of the potential barrier at the grain boundaries in perovskite SrTiO_3_ changes the low-temperature dependency of the bulk material’s electrical conductivity. By sintering samples in a reducing environment of increasing strength, we produced La_0.08_Sr_0.9_TiO_3_ (LSTO) ceramics that gradually change their electrical conductivity behavior from thermally activated to single-crystal-like, with only minor variations in the Seebeck coefficient. Imaging of the surface potential by Kelvin probe force microscopy found lower potential barriers at the grain boundaries in the LSTO samples that had been processed in the more reducing environments. A theoretical model using the band offset at the grain boundary to represent the potential barrier agreed well with the measured grain boundary potential dependency of conductivity. The present work showed an order of magnitude enhancement in electrical conductivity (from 85 to 1287 S cm^–1^) and power factor (from 143 to 1745 μW m^–1^ K^–2^) at 330 K by this modulation of charge transport at grain boundaries. This significant reduction in the impact of grain boundaries on charge transport in SrTiO_3_ provides an opportunity to achieve the ultimate “phonon glass electron crystal” by appropriate experimental design and processing.

## Introduction

Grain boundaries exist in all polycrystalline materials and have significant impact on, and may dominate, the overall structural and functional properties of the material.^1−4^ The modulation of grain boundaries’ characteristics (e.g., grain size, local elementary composition, chemical state, etc.) provides an opportunity to tune bulk properties including mechanical strength,^5^ electronic and ionic conductivity,^3,6−8^ photovoltaic efficiency,^4^ and thermal conductivity.^9^ Recently, interest has grown in the optimization and enhancement of thermoelectric properties of inorganic compounds via grain boundary (GB) engineering.^1,3,6,7^

Thermoelectric materials that can generate electricity efficiently from waste heat are of considerable interest in the development of future sustainable society.^10−13^ Particularly, in the forthcoming age of internet of things enabled by the 5G network, the thermoelectric generator that operates at room or near room temperatures can (i) convert the low-grade waste heat (30–250 °C) generated by electronic devices to electricity and (ii) power up wearable and mobile sensors using human body heat.^14−16^ For thermoelectric materials, the energy conversion efficiency is evaluated by the dimensionless figure of merit (*ZT*), which is determined by the equation *ZT* = (*S*^2^σ/κ)*T*, where *S*, σ, κ, and *T* are the Seebeck coefficient, electrical conductivity, thermal conductivity, and absolute temperature, respectively; thus, we need to maximize charge transport and minimize thermal transport.^11^ The presence of grain boundaries affects both the power factor (*S*^2^σ) and the thermal conductivity of bulk materials.^1,9,17^ For some materials, such as SrTiO_3_ and Mg_3_Sb_2_, the buildup of electrostatic potential at the GB acts as a barrier for charge carrier transport, limiting the overall carrier mobility, particularly at and near room temperature.^18−21^ Meanwhile, the GB interface is itself a type of lattice defect assemblage that assists in the reduction of thermal conductivity.^9,22^ These conflicting effects of grain boundaries on electrical and thermal transport present major challenges in the development of high-performance nanocrystalline thermoelectric materials operating over a range of temperatures. Therefore, modulation of grain boundaries, which can reduce their impact on charge carrier transport while retaining their ability to suppress lattice thermal conductivity, is in demand.

Recent work on n-type SrTiO_3_-based thermoelectric ceramics showed that the thermally activated behavior of low-temperature electrical conductivity disappeared with the use of certain processing conditions (temperature, oxygen partial pressure, etc.) and/or after incorporation of additives (e.g., graphene).^3,18,22,23^ The analysis of energy- and carrier concentration-independent weighted mobility showed that charge transport in polycrystalline SrTiO_3_ can approach that of a single crystal.^7^ Generally, this single-crystal-like electrical conductivity at low temperature is attributed to the decrease in GB resistance with the reduction of the built-in potential barrier at the grain boundaries.^3,6,7,18^ Localized changes in GB chemistry and structure (elementary composition, chemical state, etc.) are thought to be the primary reasons for the reduction of the potential barrier at the grain boundaries.^3^ However, to date, there has been no direct evidence, either experimental or theoretical, that links the GB potential with the change in temperature dependency of conductivity in thermoelectric materials.

A recently developed two-phase model,^1^ which treated the GB as a separate phase with a band offset from the neutral grain phase, was able to capture the measured temperature-dependent electrical conductivity in Mg_3_Sb_2_-based compounds. The band offset of the GB phase acted as a potential barrier for carrier transport. This two-phase model showed good agreement with the grain size-dependent conductivity in Mg_3_Sb_2_-based compounds,^1^ as the contribution of GB resistance becomes much less dominant at larger grain sizes. However, perovskite SrTiO_3_-based ceramics, with comparably small grain sizes (0.5–5 μm) exhibited distinctly different temperature dependencies of conductivity, showing either thermally activated or single-crystal-like behavior.^22,24^ This inconsistency in charge transport in SrTiO_3_-based ceramic with similar grain sizes strongly indicates that the intrinsic GB properties (e.g., built-in potential) vary from sample to sample, even for samples with a similar stoichiometry.

In this work, by combining experimental results with theoretical modeling, we directly show that the modulation of the potential barrier at grain boundaries effectively switches the low-temperature conductivity of La-doped SrTiO_3_ from thermally activated to single-crystal-like behavior. We begin with the prediction of temperature-dependent electrical conductivity using the recently developed two-phase model; by lowering the magnitude of the band offset at the GB (i.e., height of the potential barrier), the low-temperature electrical conductivity changes from thermally activated to single-crystal-like behavior. We experimentally modulated the GB properties of La_0.08_Sr_0.9_TiO_3_ (LSTO) ceramic by control of processing conditions. The as-prepared LSTO ceramic samples showed a gradual change in low-temperature electrical conductivity from the thermally activated to single-crystal type, with an order of magnitude increase in electrical conductivity and power factor at 330 K. The experimental results fit well with the two-phase model, showing good agreement for the dependency of electrical conductivity on GB potential. Moreover, the reduction in GB potential was confirmed by imaging the surface and analysis of local potential by Kelvin probe force microscopy (KPFM).

## Experimental Section

### Materials

The starting powders of TiO_2_ (>99.9%) and SrCO_3_ (>99.9%) were obtained from Sigma-Aldrich (Gillingham, Dorset, UK). La_2_O_3_ powder (>99.99%) was obtained from PI-KEM (Magnus, Tamworth, UK). Isopropanol was supplied by Sigma-Aldrich (Gillingham, Dorset, UK), and the graphene nanoplatelets (GNPs), grade M25, were obtained from XG science (Lansing, USA).

### Preparation of La_0.08_Sr_0.9_TiO_3_ (LSTO) Powder

The A-site deficient La_0.08_Sr_0.9_TiO_3_ ceramic powder was prepared by a solid-state reaction approach. The starting powders of TiO_2_, SrCO_3_, and La_2_O_3_ were weighed according to the stoichiometry before mixing. The La_2_O_3_ powder was calcined in air at 1173 K for 6 h to remove moisture before weighing. After wet milling in isopropanol for 24 h using zirconia ball milling media, the well-mixed powders were dried at 90 °C for 24 h. The solid-state reaction to form LSTO was conducted in an alumina crucible at 1473 K in air for 8 h. The as-prepared LSTO powder was subjected to planetary milling (Retsch planetary ball mill PM 100) at 350 rpm for 4 h to reduce the average particle size to ∼590 nm.

### Sintering of LSTO Ceramics

The bulk LSTO ceramics were prepared by conventional pressureless sintering. A uniaxial press was used to compact the LSTO powders into green body pellets 15 or 20 mm in diameter and 5 mm in height. The as-formed pellets were densified at 1700 K under three types of conditions with an increasingly strong reducing environment, namely, (1) Ar-H_2_-5%, (2) Ar-H_2_-5% and sacrificial carbon powder but not in direct contact with the LSTO green body, and (3) Ar-H_2_-5% and sacrificial carbon powder bed with the LSTO green body embedded; these three samples are labeled LSTO-H_2_, LSTO-H_2_-C, and LSTO-H_2_-in-C, respectively. To create more oxygen vacancies, the sintering time (24 h) for the LSTO-H_2_-C and LSTO-H_2_-in-C samples is longer than that of LSTO-H_2_ (12 h). The oxygen scavenging carbon powder bed is made of the aforementioned LSTO power + 5 wt % GNPs. After sintering, the as-formed pellets were cooled in the corresponding environments to room temperature and then cut into bars and discs of appropriate sizes for detailed characterization.

### Characterization

The density (ρ) of the LSTO ceramic was determined using the Archimedes method. The crystal structure and lattice parameters were characterized by X-ray diffraction (XRD) using a Philips X’Pert diffractometer with the Cu Kα source (λ_Cu Kα_ = 1.540598 Å). A continuous scan between 20 and 100° was recorded using 0.0167° step size and a dwell time of 6 s per step. X’Pert HighScore and TOPAS software were used for phase identification and Rietveld refinement. The grain size was undertaken on polished surfaces by scanning electron microscopy (SEM, TESCAN MIRA3 SC FEG-SEM), and the linear intercept method was used to determine the average grain size.^25^ X-ray photoelectron spectra (XPS) were collected with a Kratos Axis Ultra spectrometer using monochromatic Al Kα radiation (*E*_source_ = 1486.69 eV). CasaXPS software was used for deconvolution of the Ti 2p core level with a Shirley-type background. Atomic force microscopy (AFM) and KPFM were performed using a JPK NanoWizard 4 XP NanoScience atomic microscope equipped with a Kelvin probe microscopy module. The images were recorded using a Pt–Ir-coated silicon probe (SCM-PIT-V2, Bruker).

### Thermoelectric Measurements

A ULVAC ZEM-3 system was used to simultaneously determine electrical conductivity and Seebeck coefficients; the measurements were performed at temperatures from 300 to 900 K in a low-pressure helium atmosphere. This inert atmosphere with low oxygen partial pressure, together with the medium–high temperature limit of 900 K, ensures the stability of materials’ properties during measurements.^26,27^ Thermal diffusivity (*D*) was determined using the laser flash method with a Netzsch LFA-457 laser flash apparatus in an argon atmosphere. Differential scanning calorimetry (Netzsch DSC 404 F1 Pegasus) was used to measure the heat capacity (*C*_*p*_); the measurements were performed in an argon atmosphere. The thermal conductivity (κ) was obtained from κ = *D*ρ*C*_*p*_.

## Results and Discussion

**Figure 1:**
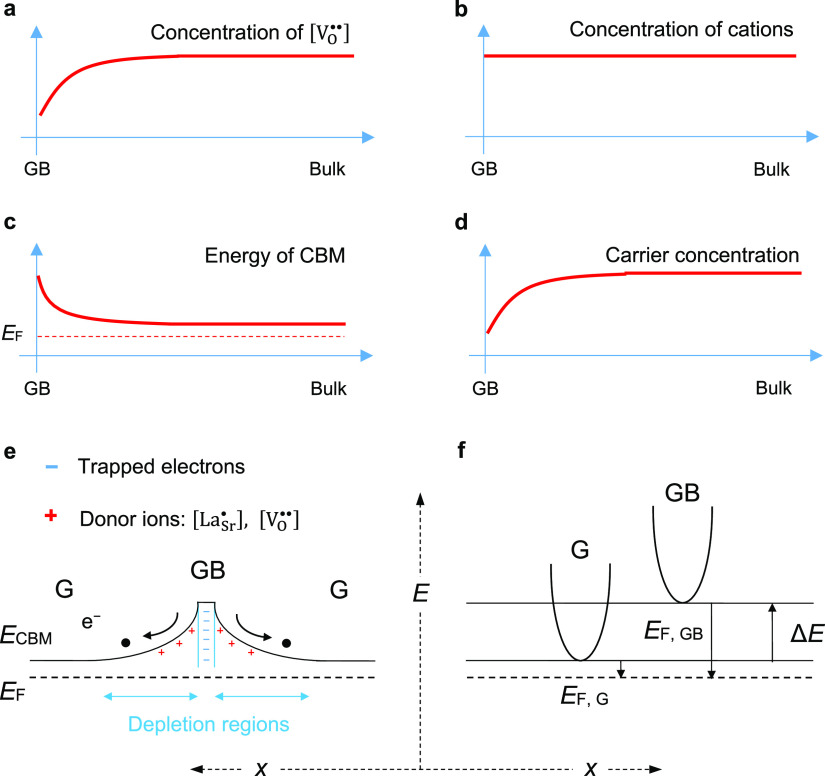
Diagrams showing that (a) concentration of oxygen vacancy is lower near the GB compared with the bulk grain, (b) concentrations of cations remain across the GB, (c) CBM bends upward because of the equilibrium of the Fermi energy level, and (d) reduced concentration of the free carrier at the vicinity of the GB. (e) Schematic illustration of the potential barrier across the GB, G represents a neutral grain and the black dots and arrows represent the transport of free electrons being inhibited by the GB barrier. (f) Schematic illustration of the two-phase model for the band offset (Δ*E*) in the GB phase.

Figure 1a–d illustrates the way that a negatively charged GB forms and behaves as a potential barrier for electron transport in SrTiO_3_-based materials. Based upon Snyder’s work,^3,7^ the formation of a potential barrier in n-type SrTiO_3_ can be ascribed to the reduction of the concentration of the dominant point defect (oxygen vacancies) near the GB (Figure 1a). Literature results from molecular dynamic simulation also indicate the depletion of oxygen vacancies in regions near dislocation cores,^28^ which are analogue lattice defects to the grain boundaries. On the assumption that the concentrations of cations are constant across the GB (Figure 1b), the depletion of positively charged oxygen vacancies (electron-donating defects) near grain boundaries compared to bulk grains induces the difference in Fermi energy levels, promoting electron transfer from the grain to the GB to maintain the equilibrium of the Fermi energy level. This charge transfer leads to a negative potential at the GB region, which alters the local electronic structure at the GB by bending the conductive band upward (Figure 1c).^1,3,29^ Energy band diagrams of two grains and their boundary region before and after contact are illustrated in Figure S1a,b, respectively. The trapped electrons at the GB results in much less free carriers in the vicinity of the GB and thus greater resistance compared to the bulk neutral grain (Figure 1d). Figure 1e depicts the overall principle of a GB potential in n-type La-doped SrTiO_3_ acting as a barrier for majority carrier (i.e., electrons) transport. In addition, the grain boundaries with lattice mismatch act as scattering centers for carrier transport. The combination of a potential barrier and lattice defects at the grain boundaries leads to compromised electrical conductivity compared to that of the bulk neutral grain. The two-phase model treats the GB as a secondary phase with (i) a band offset (Δ*E*) from the bulk grain phase (Figure 1f), representing the band bending, and (ii) a different carrier scattering mechanism from the bulk grain phase, mimicking carrier scattering, because of the lattice mismatch.

In detail, the Fermi energy level of a GB phase (*E*_F,GB_) measured from the band edge (CBM: conductive band minimum) is defined by the band offset to be^1^
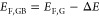
1where *E*_F,G_ is the Fermi energy level of the grain phase measured from the CBM, positive for free carrier energy that is higher than the CBM energy level.

Since the band offset Δ*E* also depends on the doping level of the material, an empirical, linear form of the band offset function can be used to describe the energy dependency of Δ*E*
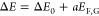
2where Δ*E*_0_ is the reference band offset at *E*_F,G_ = 0; it defines the magnitude of the band offset and therefore acts as a modeling parameter in this work; the coefficient *a* is an empirical parameter which determines the energy dependency of Δ*E*.

With a known Fermi energy level, the electrical conductivity (σ) and Seebeck coefficient (*S*) of the grain and GB phases can be calculated by the well-established carrier transport equations^30^
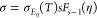
3
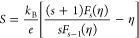
4where the transport coefficient σ_*E*_0__(*T*) is a temperature-dependent but energy-independent coefficient that determines the magnitude of electrical conductivity; *T* is the absolute temperature, *s* is the transport parameter related to the carrier scattering mechanism; η is the reduced Fermi energy level (η = *E*_F_/*k*_B_*T*); *k*_B_ is the Boltzmann constant; *e* is the elementary charge, and *F*_*i*_(η) is the Fermi–Dirac integral
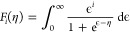
5where ϵ is the reduced particle energy (ϵ = *E*/*k*_B_*T*; *E*: particle energy).

For the neutral grain phase of SrTiO_3_ compounds, acoustic phonon scattering (*s* = 1) is assumed to be the dominant carrier scattering mechanism,^7,22^ with a crystalline metallic-type temperature dependency of the transport coefficient (σ_*E*_0_,G_(*T*) ∝ 1/*T*; the subscript G denotes the grain phase). Earlier studies suggest that the donor concentration (point defects: [La_Sr_^•^] or [V_O_^••^]) dependency of carrier mobility in single-crystal SrTiO_3_ only becomes significant at low temperatures (e.g., <150 K).^17,31^ The present work is focusing on room temperature and above, and thus, the ionic impurity scattering is not included as a scattering source in bulk neutral grain. We verified this assumption by fitting literature data for carrier transport for La- or Nb-doped SrTiO_3_ single crystals,^32^ using the value of σ_*E*_0_,G_ at 300 K as a fitting parameter. The modeled results with σ_*E*_0_,G_ = 900 S cm^–1^ at 300 K (Figure S2) agree well with the reported temperature dependency of electrical conductivity, as well as the log |*S*| – log σ plot. Hence, *s* = 1 and σ_*E*_0_,G_(*T*) ∝ 1/*T* with a value of 900 S cm^–1^ at 300 K were used as universal modeling parameters for the neutral grain phase in this work. For the GB phase with space charge and lattice mismatch, an ionized-impurity scattering model (*s* = 3; σ_*E*_0_,GB_(*T*) ∝ *T*^3^, the subscript GB denotes the GB phase) was used,^1^ with σ_*E*_0_,GB_ = 0.15 S cm^–1^ at 300 K. Table 1 shows the optimized modeling parameters for our materials.

**Table 1:** Modeling Parameters for La_0.08_Sr_0.9_TiO_3−δ_ (LSTO) Prepared with Different Sintering Conditions

		band offset function		transport coefficient (300 K)	
sample	*t*_GB_^a^	*a*	Δ*E*_0_ (meV)	σ_*E*_0_,G_ (S cm^–1^)	σ_*E*_0_,GB_ (S cm^–1^)
LSTO-H_2_	0.001 (1.75 μm)	0.3	100	900	0.15
LSTO-H_2_-C	0.0005 (3.69 μm)		75		
LSTO-H_2_-in-C	0.0005 (2.67 μm)		10		

a The average grain size of the sample is shown in the parenthesis.

A simple series circuit model was used to calculate the overall charge transport behavior; the overall electrical conductivity σ was obtained from
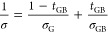
6where *t*_GB_ is the size fraction of the GB phase in the ceramic. The value of *t*_GB_ is proportional to the grain size and thus can be estimated from microstructure characterization. This estimation of *t*_GB_ from the grain size assumes that the thickness of the carrier-depleted region is consistent for different samples, that is, the thickness of the carrier depletion region is independent of the magnitude of the potential barrier. This assumption is reasonable as the SrTiO_3_-based ceramics developed for thermoelectric applications have high donor concentrations, typically ∼10^20^ cm^–3^; therefore, the charge screening length should be similar. Although a more accurate description of a real GB should correlate the thickness of the depletion region to the magnitude of the potential barrier,^33^ we found that the current approximation is sufficiently good to fit the experimental results.

For the overall Seebeck coefficient *S*, in view of the small fraction of the GB phase in the ceramic (*t*_GB_ < 0.001), we followed the recent work of Kuo et al.^1^ and assumed
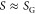
7where *S*_G_ is the Seebeck coefficient of the bulk grain; see detailed explanations in the Supporting Information and Figure S3. Note that the energy filtering effect of GB barriers on Seebeck coefficients only becomes prominent for nanostructured materials with grain sizes <50 nm or the size fraction of the barrier phase being high.^34,35^ The present work focuses on SrTiO_3_-based ceramics that have grain sizes over 1 μm, and thus, their Seebeck coefficients should be dominated by carrier concentration. The assumption of *S* ≈ *S*_G_ allowed extraction of the reduced Fermi energy level η of the grain phase, using eq 4, from the experimentally measured overall Seebeck coefficient of the ceramic. In turn, this allows calculation of the reduced Fermi energy level of the GB phase using eq 1 and 2. Subsequently, the carrier transport characteristic of the polycrystalline SrTiO_3_ was modeled with only one variable, Δ*E*_0_.

**Figure 2:**
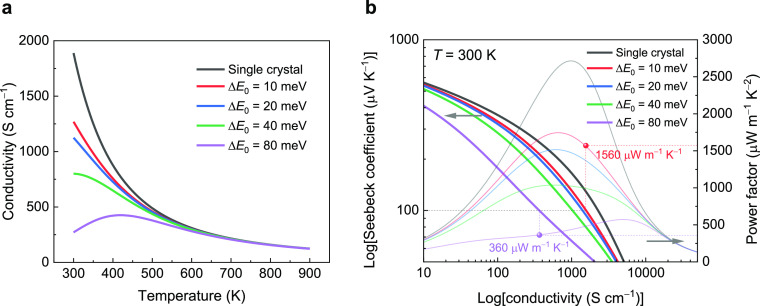
Theoretical predictions for GB-dominated charge transport in SrTiO_3_ ceramics using the two-phase model. (a) Modeled temperature dependency of electrical conductivity for SrTiO_3_ ceramics with different reference band offset values (Δ*E*_0_) and a fixed *t*_GB_ value of 0.0005; other parameters (*a*, σ_*E*_0_,G_, and σ_*E*_0_,GB_) are kept at the universal values shown in Table 1. (b) Simulated log |*S*| – log σ plots (thick solid lines) and plots of the power factor as a function of log σ (feint lines) at room temperature (300 K) for SrTiO_3_ ceramics with different values of Δ*E*_0_. The modeled results for a single crystal (*t*_GB_ = 0; solid black line) are added for reference purposes.

To demonstrate the approach, we modeled the impact of Δ*E*_0_ on the temperature dependencies of electrical conductivity (Figure 2a); the set of Seebeck coefficients for determining the reduced Fermi energy level of the grain phase was obtained from the LSTO-H_2_ sample that will be discussed later. Obviously, with the decrease in Δ*E*_0_ from 80 to 10 meV, the temperature dependency of conductivity at low temperatures (300–450 K) switches from a thermally activated type to a metallic type. The dramatically enhanced conductivity suggests high power factor values, as the increase in conductivity is due to the change in GB property rather than a change in the carrier concentration of the neutral grain phase (i.e., Seebeck coefficients remain unchanged). The plots of modeled log |*S*| – log σ and power factor—log σ (Figure 2b) clearly illustrate the enhancement in the powder factor by reduction of GB barrier heights. For instance, with a Seebeck coefficient of 100 μV K^–1^, the reduction of Δ*E*_0_ from 80 to 10 meV leads to the enhancement of the power factor from 360 to 1560 μW m^–1^ K^–2^ (labeled in Figure 2b).

**Figure 3:**
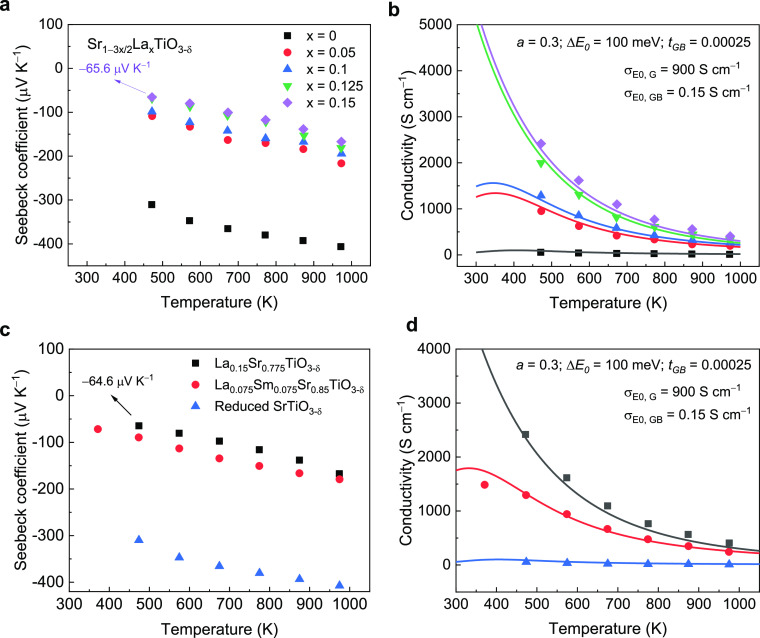
Fitting of the measured carrier transport results from the literature using the two-phase model. Experimental data points are displayed as symbols, and the modeled results are shown as solid lines. Data points for Seebeck coefficients (a) and electrical conductivity (b) are from the work of Lu et al.^36^ Data points for the Seebeck coefficient (c) and electrical conductivity (d) are from Boston et al.^18^

We then fitted literature data for electrical conductivity for SrTiO_3_-based ceramics,^18,36^ using the two-phase model. Typical fitting results are presented in Figure 3. We found that a set of universal fitting parameters (*a* = 0.3, Δ*E*_0_ = 100 meV, *t*_GB_ = 0.00025, σ_*E*_0_,G_ = 900 S cm^–1^, and σ_*E*_0_,GB_ = 0.15 S cm^–1^) is sufficient to provide good agreement with the experimental results. In addition, for La_*x*_Sr_1–*y*_TiO_3_ with the medium–high doping level (*x* = 0.125 to 0.15), the high electrical conductivity due to high carrier concentration overwhelmed GB resistance at low temperatures, leading to a metallic-type conductivity. However, this way of achieving high conductivity at low temperatures, by heavy doping, has the disadvantage that Seebeck coefficients are severely reduced. In fact, a small Seebeck coefficient, down to ∼65 μV K^–1^ at 470 K as shown in Figure 3a,c, is required to give the neutral-grain sufficiently high electrical conductivity to overwhelm the GB resistance at room temperature (Figure 3b,d).

The current literature lacks results from a set of pure SrTiO_3_-based samples that have the same stoichiometry and comparable Seebeck coefficients, but distinctly different electrical conductivities, to allow a detailed study of GB effects. Nevertheless, an earlier report,^17^ together with recent investigations,^3,18,22^ of SrTiO_3_-based electronic ceramics implies that it should be possible to experimentally modulate the GB property by the control of processing conditions (temperature, oxygen partial pressure, etc.). The change in processing conditions, such as a higher temperature and/or more reducing atmosphere with lower oxygen partial pressure, lead to a transformation of low-temperature conductivity from thermally activated to a metallic type.^18,37^ This change in conductivity behavior is suspected to be predominantly due to the modulation of grain boundaries (e.g., a localized increase in concentration of oxygen vacancies).^17^

**Figure 4:**
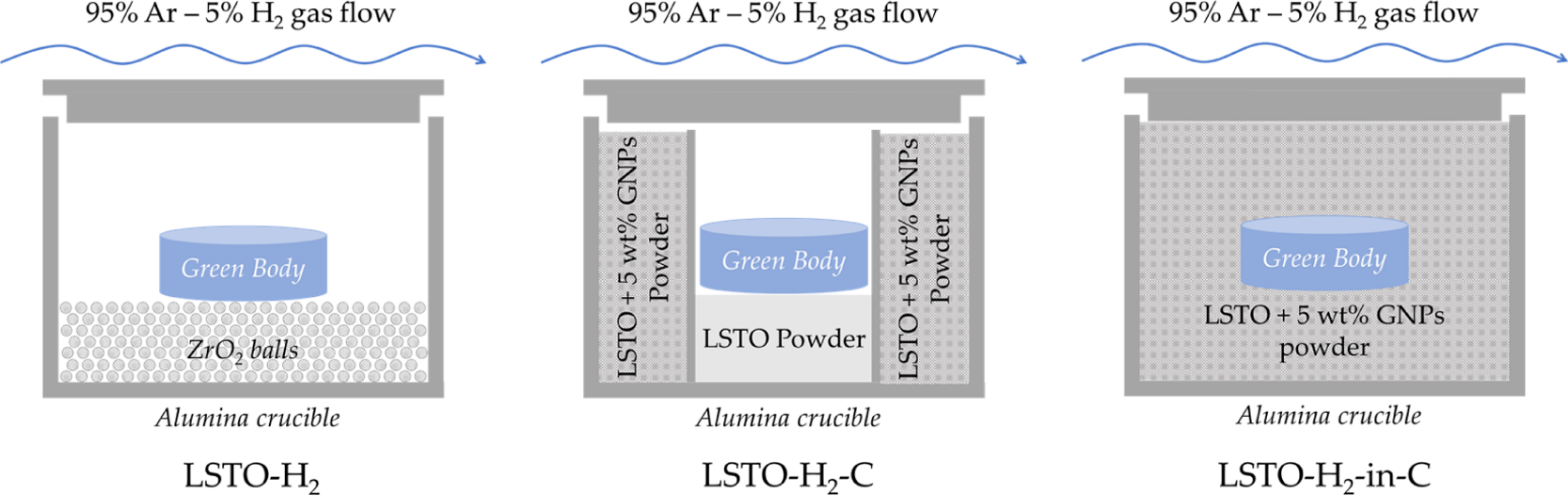
Schematic illustrations for the sintering of LSTO ceramics under different conditions with an increasingly strong reducing environment from left to right. The oxygen scavenging sacrificial carbon powder bed is made of LSTO + 5 wt % GNPs.

Therefore, we experimentally modulated the GB properties (e.g., oxygen vacancy concentration) of La_0.08_Sr_0.9_TiO_3_ (LSTO) ceramic via control of the reducing strength of the sintering environment (indirect control of oxygen partial pressure). Sample preparation details are illustrated in Figure 4 and described in the Experimental Section. The relative densities for the sintered LSTO-H_2_, LSTO-H_2_-C, and LSTO-H_2_-in C samples are 94.0, 96.0, and 89.3%, respectively.

**Figure 5:**
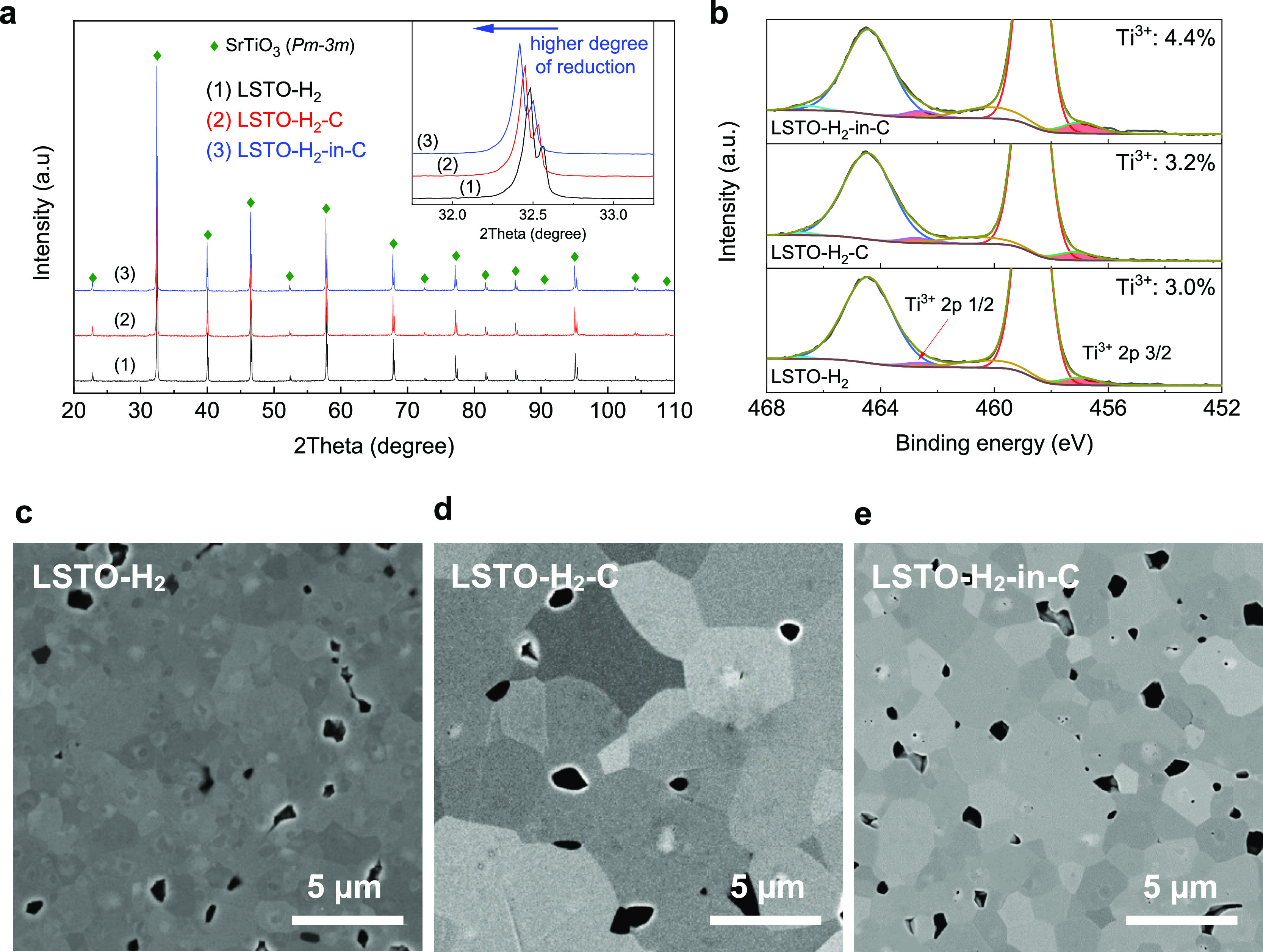
Characterization of the LSTO ceramics sintered under three different conditions with an increasingly strong reducing environment. (a) XRD pattern for the three LSTO samples. (b) XPS Ti 2p core levels for the three LSTO samples. (c–e) SEM micrographs showing grain sizes of the LSTO-H_2_, LSTO-H_2_-C, and LSTO-H_2_-in-C samples, respectively.

XRD patterns collected from the three LSTO samples (Figure 5a) show a cubic perovskite structure with *Pm*3̅*m* space group symmetry. The decrease in diffraction angle for the samples prepared in more reducing environments (inset of Figure 5a) suggests an increase in the lattice parameter (Table 2). This increase corresponds to an increasing fraction of the small-sized Ti^4+^ (60.5 pm) being converted to relatively larger-sized Ti^3+^ (67 pm).^23^ XPS further confirmed the increasing degree of reduction of the LSTO samples from the more reducing environments. The deconvolution of Ti 2p core levels (Figure 5b) leads to quantization of the concentration of Ti^3+^ as a fraction of total Ti, that is, (Ti^3+^/Ti); the Ti^3+^ concentrations for the LSTO-H_2_, LSTO-H_2_-C, and LSTO-H_2_-in-C are 3.0, 3.2, and 4.4%, respectively. This enhancement in the Ti^3+^ concentration implies a higher concentration of oxygen vacancies according to
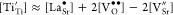
8

**Table 2:** Carrier Concentration Estimated from the Ti^3+^/Ti and the Corresponding Drift Mobility and Effective Mass at 330 K

sample	*a* (Å)	Ti^3+^/Ti (%)	*n* (10^20^ cm^–3^)	σ (S cm^–1^)	*S* (μV K^–1^)	μ (cm^2^ V^–1^ s^–1^)	m*/*m*_0_^a^
LSTO-H_2_	3.9080	3.0	5.02	85 ± 4	–129 ± 9	1.1	3.7
LSTO-H_2_-C	3.9087	3.2	5.39	492 ± 25	–116 ± 8	5.7	3.5
LSTO-H_2_-in-C	3.9095	4.4	7.36	1288 ± 64	–115 ± 8	10.9	4.2

a Free electron mass (*m*_0_).

The determination of Ti^3+^ concentration also allows the estimation of carrier concentration (*n*) by^38^
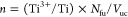
9where *N*_fu_ is the number of formula per unit cell (1 for SrTiO_3_) and *V*_uc_ is the volume of the unit cell. The calculated results are displayed in Table 2. The LSTO-H_2_-in-C shows the highest carrier concentration of 7.36 × 10^20^ cm^–3^.

**Figure 6:**
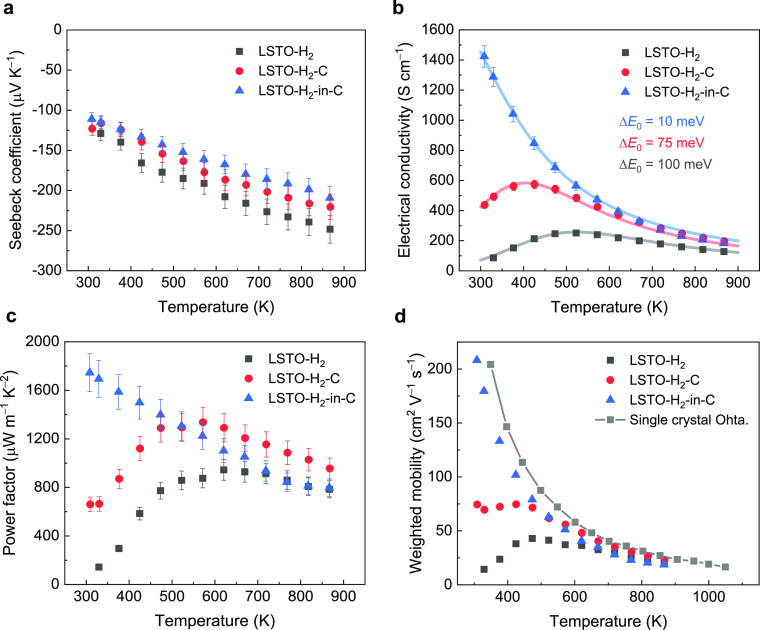
Temperature-dependent electric charge transport properties of the as-prepared LSTO ceramic samples; experimental data are represented by solid symbols. (a) Seebeck coefficient and (b) electrical conductivity; solid lines are from the model, using the parameters listed in Table 1; the simulated curves well capture the evolution of electrical conductivity with temperature. (c) Power factor and (d) weighted mobility; the weighted mobility of single-crystal La-doped SrTiO_3_ calculated from the literature data^32^ is included for comparison purposes.

Figure 6 displays the temperature-dependent carrier transport properties for the three LSTO ceramic samples. The absolute value of the Seebeck coefficient (Figure 6a) is slightly lower for the samples sintered in the more reducing environments, corresponding to the higher carrier concentrations implied by the Ti^3+^ concentration (Table 2). In contrast to the relatively small variation in the Seebeck coefficient, the three LSTO samples exhibit distinctly different electrical conductivities, particularly at temperatures below 500 K (Figure 6b). For example, at 330 K, there is over 1 order of magnitude difference in electrical conductivity between the LSTO-H_2_ (85 S cm^–1^) and the LSTO-H_2_-in-C (1287 S cm^–1^) samples. Considering the variation of density between these three samples, we estimated their effective conductivities at 330 K following a published approach based on Maxwell equations,^39^ see details in the Supporting Information and Table S1. The results show that the effective electrical conductivity is higher than the experimental values, and the conductivity increase due to correction is more pronounced for the samples containing a higher degree of porosity.

In addition to distinct differences in the magnitude of electrical conductivity, the as-prepared samples also show very different temperature dependencies, particularly at low temperature. Both of the LSTO-H_2_ and LSTO-H_2_-C samples show a typical GB-dominated, thermally activated conductivity, that is, at low temperatures, the electrical conductivity increases, reaches a maximum, and then decreases at higher temperatures. Due to the larger grain size of LSTO-H_2_-C and plausibly lower GB potential barrier compared to LSTO-H_2_, the LSTO-H_2_-C has higher electrical conductivity at low temperatures and peak electrical conductivity at a lower temperature (smaller thermal energy needed to overcome the GB resistance). More interestingly, although LSTO-H_2_-in-C has a smaller grain size (2.67 μm) than that of the LSTO-H_2_-C (3.69 μm), the LSTO-H_2_-in-C sample exhibits a metallic-type (i.e., single-crystal-like) electrical conductivity in the measured temperature range from 300 to 900 K, with the highest conductivity of up to 1423 S cm^–1^ at ∼300 K. This switch of temperature dependency is primarily due to the change in the grain boundaries’ property (barrier height) rather than the grain size.

To provide further insight into the GB processes, we first calculated the electrical conductivity of the neutral grain phase using eq 3 and the reduced Fermi energy level η extracted from the measured Seebeck coefficients (Figure 6a) according to eq 4. The calculated conductivities of the neutral grain phase for the LSTO-H_2_, LSTO-H_2_-C, and LSTO-H_2_-in-C samples are quite similar, particularly the latter two (Figure S4). This decoupling of the neutral-grain-phase electrical conductivity from the measured overall conductivity directly indicates the dominance of grain boundaries in carrier transport in polycrystalline SrTiO_3_-based ceramics. We subsequently fitted the measured electrical conductivity data with the two-phase model (eq 6), using parameters listed in Table 1; the reduced Fermi energy level of the GB phase was calculated using eq 1 and the band offset function (eq 2), with Δ*E*_0_ as the only fitting parameter. The value of *t*_GB_ was chosen according to the average grain size from SEM images (Figure 5c–e) and empirically according to the literature,^1^ as discussed above, and is therefore considered as a “known” parameter. The solid lines displayed in Figure 6b are from the two-phase model; the simulated results fit well to the measured electrical conductivity. The Δ*E*_0_ values that lead to the best fit of the measured conductivity data are 100, 75, and 10 meV for LSTO-H_2_, LSTO-H_2_-C, and LSTO-H_2_-in-C samples, respectively.

The power factor (*S*^2^σ) exhibits similar temperature dependency as that of the electrical conductivity (Figure 6c). The LSTO-H_2_-in-C has the highest power factor value of 1745 μW m^–1^ K^–2^ at room temperature, an order of magnitude higher than that of the LSTO-H_2_ sample (143 μW m^–1^ K^–2^) at 330 K. In addition, the LSTO-H_2_-C also shows a high power factor value of 1337 μW m^–1^ K^–2^ at 570 K. These power factor values are among the highest values reported for SrTiO_3_-based compounds (Table S2).^6,18,20−24,36,38,40−44^

We further computed the weighted mobility (μ_w_) of the LSTO samples from the measured electrical conductivity and Seebeck coefficients using the approach reported recently by Snyder et al.^7,45^ In detail, the LSTO ceramic was treated as a homogeneous phase with acoustic phonon scattering as the dominating scattering mechanism (transport parameter *s* = 1), the reduced Fermi energy level η at each temperature was extracted from the Seebeck coefficients using eq 4, and then, the temperature-dependent transport coefficient σ_*E*_0__(*T*) was computed according to eq 3. The determination of the transport coefficient σ_*E*_0__(*T*) then leads to the calculation of weighted mobility by
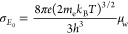
10where *m*_e_ is the free electron mass and *h* is the Plank constant. This weighted mobility is temperature-dependent but energy- and carrier concentration-independent and allows a direct comparison of carrier mobility for samples at various doping levels. The comparison of weighted mobility derived from the literature data for SrTiO_3_-based single crystals^32^ shows similar values of weighted mobility, regardless of the doping concentration (Figure S5). Figure 6d shows the computed μ_w_ for the three LSTO samples prepared in the present work. Significant enhancement of μ_w_ is obvious for the LSTO prepared in the most reducing environment; it has the smallest Δ*E*_0_ value from the two-phase model. Excitingly, the LSTO-H_2_-in-C sample with a metallic-type electrical conductivity at the measured temperature range exhibits a weighted mobility approaching that of the single crystal (Figure 6d).

The carrier concentration *n* determined from compositional data by eq 9 also allows the estimation of drift mobility (μ) from the measured electrical conductivity using the Drude–Sommerfeld free-electron model
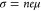
11

Furthermore, with the parabolic band and energy-independent scattering approximation, the carrier effective mass (m*) was estimated from the measured Seebeck coefficients by^38^
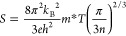
12

The estimated values of μ and m* at 330 K are presented in Table 2. It is interesting to note that the best performing LSTO-H_2_-in-C sample shows the highest effective mass (m*/*m*_0_ = 4.2) and highest drift mobility (μ = 10.9) among the three measured samples. These values are comparable to literature values reported for mobility (μ = 9.2) and effective mass (m*/*m*_0_ = 6.0) for a single crystal with a similar carrier concentration (*n* = 6.8 × 10^20^ cm^–3^) at room temperature.^32^

**Figure 7:**
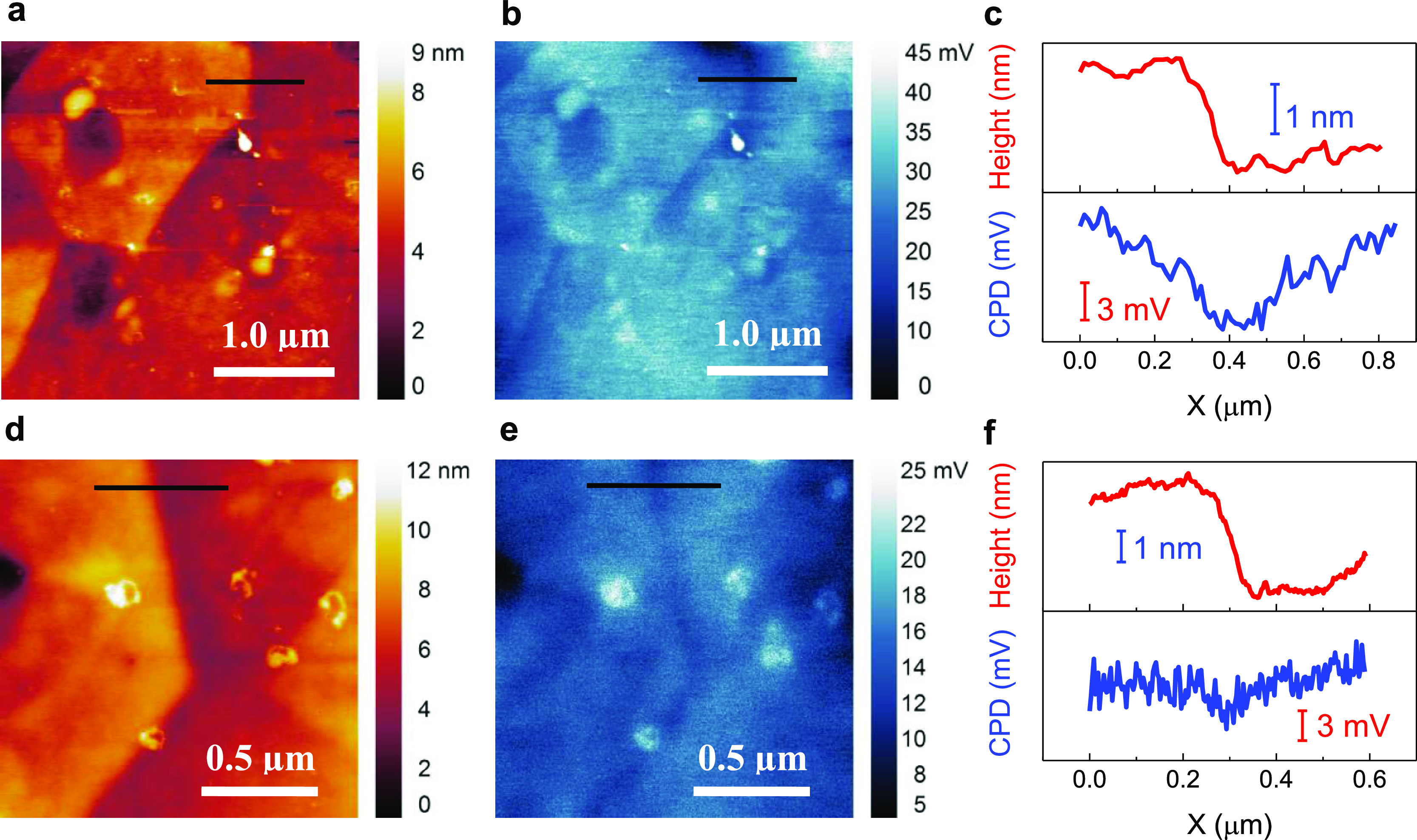
AFM and KPFM characterization for the LSTO-H_2_ and LSTO-H_2_-in-C samples. (a) AFM height and (b) the corresponding CPD images for the LSTO-H_2_ sample with high GB potential; (c) extracted height (upper panel) and CPD (lower panel) profiles for the black lines marked in (a,b), respectively. (d) AFM height and (e) the corresponding CPD images for the LSTO-H_2_-in-C sample with low GB potential; (f) the extracted height (upper panel) and CPD (lower panel) profiles for the black lines marked in (d,e), respectively.

The carrier transport and modeling results strongly indicate a reduction of the height of potential barriers at grain boundaries. To verify this reduction of the potential barrier, we used KPFM, also known as scanning surface potential microscopy, to characterize the contact potential difference (CPD) across the grain boundaries. The AFM height images of the sample surface were collected simultaneously during the KPFM measurement. Figure 7 presents the height and CPD images collected from the LSTO-H_2_ (Figure 7a,b) and LSTO-H_2_-in-C (Figure 7d,e) samples. The grain boundaries are clearly distinguishable in both the height and CPD images due to the height variation of adjacent grains and the existence of potential barriers at the grain boundaries, respectively. The height profiles (Figure 7c,f) extracted from the lines marked in Figure 7a,d indicate a well-defined stair feature, showing a height variation of around 2 nm between the adjacent grains. In contrast, the profiles of CPD do not follow that of the topography but exhibit a valley at the GB region, suggesting negatively charged grain boundaries as expected.

More importantly, the magnitude of this negatively charged potential barrier at the GB clearly differs between the two measured samples, ∼6 mV for the LSTO-H_2_ sample and <3 mV for the LSTO-H_2_-in-C. The smaller magnitude of negative potential at the GB of LSTO-H_2_-in-C compared to that of the LSTO-H_2_ sample correlates well with differences in their temperature dependency of conductivity and the Δ*E*_0_ values from the two-phase model (Figure 6b). Obviously, the measurement of surface potential by KPFM provides direct evidence that the LSTO-H_2_-in-C sample with a single-crystal-like carrier transport behavior has a smaller potential barrier at the grain boundaries than the LSTO-H_2_ with thermally activated conductivity. Nevertheless, there is a discrepancy between specific values of the measured potential barrier (several mV in scale) by KPFM and the band offset Δ*E* (10–100 meV) from the fitting of electrical conductivity with the two-phase model. This discrepancy is probably due to charge accumulation at the sample surface and the resolution limit of KPFM, causing the lowering and widening of the potential profile.^46^

**Figure 8:**
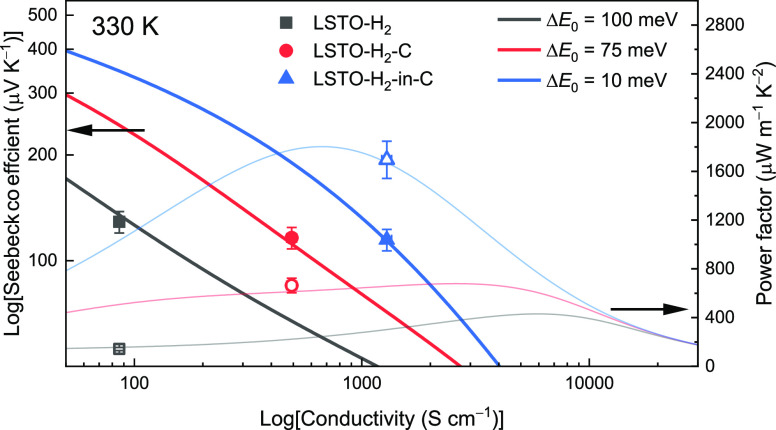
Modeled plots of (i) log Seebeck coefficients as a function of log electrical conductivity (thick solid lines) and (ii) power factor as a function of log electrical conductivity (feint lines) at room temperature (330 K) for the three LSTO ceramic samples prepared in the present work. The symbols are experimental results of log |*S*| – log σ (solid symbols) and power factor vs log σ (open symbols).

The two-phase model successfully reproduced the temperature-dependent electrical conductivity of the LSTO samples with the Δ*E*_0_ defining the magnitude of band offset as the only fitting parameter. The measurement of GB potential by KPFM confirmed the soundness of using the magnitude of the band offset (i.e., potential barrier height) at the GB as a fitting parameter. We further simulated plots of (i) log |*S*| – log σ and (ii) power factor as a function of log σ at a temperature of 330 K, using the parameters listed in Table 1. The simulated plots are presented as lines in Figure 8; the symbols are experimental values from the three LSTO samples prepared in this work. The simulated plots fit well with the experimental results. According to Figure 8, it is clear that we achieved an order of magnitude enhancement in electrical conductivity with only minor sacrifice of Seebeck coefficients by the modulation of GB properties. This conductivity enhancement further leads to an order of magnitude increase in power factor. Nevertheless, the power factor of the LSTO ceramic has not yet fully approached that of the single crystal. On the basis of the two-phase model, further enhancement of the power factor is achievable by reduction of the size fraction of GB phase *t*_GB_ and/or the increase in the transport coefficient of the GB phase σ_E_0_,GB_.

According to this significant enhancement of the power factor at room temperature, the *ZT* value of LSTO-H_2_-in-C increases by an order of magnitude to 0.07 from that of LSTO-H_2_ (0.007) at 330 K (Figure S6). To achieve a high *ZT* value, it is necessary to reduce/maintain thermal conductivity of LSTO at a relatively low level while enhancing its electrical conductivity. Ideally, the lattice contribution to the thermal conductivity should remain unchanged when the electronic transport behavior switches from a thermally activated to a single-crystal-like behavior. From the Wiedemann–Franz law, we estimated the electronic contribution (κ_electronic_) of the thermal conductivity by κ_electronic_ = *L*σ*T*, where *L* is the Lorenz number and is determined by the transport equation (single parabolic band, acoustic phonon scattering *s* = 1)
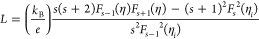
13

The reduced Fermi energy level η at each temperature was extracted from measured Seebeck coefficients according to eq 4. The lattice contribution (κ_lattice_) to the total thermal conductivity (κ_total_) is obtained by κ_lattice_ = κ_total_ – κ_electronic_.

This deconvolution of electronic and lattice contributions to the total thermal conductivity (Figure S7a,b) indicates that the higher total thermal conductivity of LSTO-H_2_-in-C (8.1 W m^–1^ K^–1^) compared to that of LSTO-H_2_ (6.9 W m^–1^ K^–1^) at 323 K is primarily due to the electronic contribution (0.8 W m^–1^ K^–1^). This much reduced impact of GB modulation on the lattice thermal conductivity compared to electrical conductivity implies an opportunity to achieve the ultimate “phonon glass electron crystal” target by grain size refinement. Earlier work demonstrated a room temperature thermal conductivity as low as 1.2 W m^–1^ K^–1^ for La-doped (10 at %) SrTiO_3_ ceramic with a grain size of 24 nm.^9^ Moreover, for nanostructured materials with a grain size of <50 nm, energy filtering effects become predominant.^34,35^ An earlier computational study indicates an opportunity to enhance both the Seebeck coefficient and power factor via the energy filtering effect by a precise control of both the barrier height and grain size, the optimum barrier height being around *k*_B_*T*, ∼26 meV at room temperature,^35^ which is close to the modeled value in this work for the LSTO-H_2_-in-C sample (10 meV). However, the creation of a sufficiently high concentration of oxygen vacancies at the grain boundaries requires high temperature and long-term annealing of LSTO in low oxygen partial pressure environments, which is in conflict with the need to limit grain growth. This challenging target might be achievable via the advancement of ceramic processing technologies and/or development of composites in which the secondary components (e.g., graphene) restrain grain growth, while facilitating the creation of oxygen vacancies at the GB.^6,7,22,40,43^

## Conclusions

We have shown that by both experimental results and modeling, the modulation of the built-in electrostatic potential at the grain boundaries of SrTiO_3_ leads to charge carrier transport approaching that of single crystals. The two-phase model successfully accounted for the GB potential as a band offset from the neutral grain, highlighting its dominant role in tuning the low-temperature dependency of electrical conductivity. The model showed good agreement with the properties of experimentally prepared LSTO samples that exhibit gradual changes in electrical conductivity behavior from the thermally activated to single-crystal type. KPFM imaging of the surface potential further confirmed the reduced GB potential for LSTO samples with single-crystal-like carrier transport behavior. The successful modulation of GB charge transport leads to an order of magnitude enhancement in the power factor value from 143 to 1745 μW m^–1^ K^–2^ at 330 K. This work provided further insights and understanding of GB effects on the electrical charge transport in polycrystalline SrTiO_3_ perovskite and guidance in the design of effective routes to further enhance performance via GB engineering.

## Supporting Information Available

Additional modeling details for Seebeck coefficients in the series circuit model; computation of porosity corrected, effective electrical conductivity; energy band diagrams of two grains and their boundary region before and after contact; fitting of the literature reported single-crystal carrier transport data with the carrier transport equations; plot of the total Seebeck coefficient as a function of *t*_GB_ in the series circuit model; decoupled electric conductivity of the neutral grain phase as a function of temperature for the LSTO samples; weighted mobility of literature-reported La- or Nb-doped single crystals; dimensionless figure of merit for the LSTO samples; and total, lattice, and electronic thermal conductivity for the LSTO samples (PDF)
