# Supplementary material for: Modulation of Charge Transport at Grain Boundaries in SrTiO3: Toward a High Thermoelectric Power Factor at Room Temperature
Source: ACS Appl Mater Interfaces. 2021 Mar 4;13(10):11879–90. doi: 10.1021/acsami.0c21699 (PMC8023519; doi:10.1021/acsami.0c21699)
Supplement: Supplementary file 1 — am0c21699_si_001.pdf [file am0c21699_si_001.pdf]

Supporting information

Modulation of Charge Transport at Grain Boundaries in $SrTiO_3$: Toward a High Thermoelectric Power Factor at Room Temperature

Jianyun Cao,[†,‡,∥] *Dursun Ekren,*[†,§,∥] *Yudong Peng,*[†] *Feridoon Azough,*[†] *Ian A. Kinloch,*[†,‡,*] *Robert Freer*[†,*]

[†] Department of Materials, University of Manchester, Oxford Road, M13 9PL, U.K.

[‡] National Graphene Institute and Henry Royce Institute, University of Manchester, Oxford Road, M13 9PL, U.K.

[§] Department of Metallurgy and Materials Engineering, Iskenderun Technical University, 31200, Iskenderun, Hatay, Turkey

[∥] These authors contributed equally to this work.

*Corresponding authors.

E-mail address: ian.kinloch@manchester.ac.uk; robert.freer@manchester.ac.uk

Seebeck coefficient in series circuit model

The overall Seebeck coefficient (S) of a series circuit model is calculated by:[1]

$$S = \frac{S_{\mathrm{G}} \frac{1 - t_{\mathrm{GB}}}{\kappa_{\mathrm{G}}} + S_{\mathrm{GB}} \frac{t_{\mathrm{GB}}}{\kappa_{\mathrm{GB}}}}{\frac{1 - t_{\mathrm{GB}}}{\kappa_{\mathrm{G}}} + \frac{t_{\mathrm{GB}}}{\kappa_{\mathrm{GB}}}} \tag{S1}$$

where the subscript G and GB represent the grain and grain boundary, respectively; t_{GB} is the size fraction of grain boundary phase in the material, κ is thermal conductivity. The value of t_{GB} is very small (< 0.001),[1] and the thermal conductivities of grain and grain boundary phase of the same order of magnitude (1~10), with similar values of S_{G} and S_{GB}. Therefore,

$$S \approx S_{\mathrm{G}} \tag{S2}$$

The plot of S versus t_{GB} is provided in **Figure S3** for reference.

Effect of porosity on electrical conductivity

The effect of porosity on the electrical conductivity can be represented by calculation of effective electrical conductivity using literature reported approach based on Maxwell equations.[2] In detail,

$$\frac{\sigma}{\sigma_0} = \frac{1-\varepsilon}{1-0.5\varepsilon} \tag{S3}$$

where; σ is the experimental electrical conductivity, σ_0 is the effective electrical conductivity and ε is the porosity of the material which can be calculated using the following equation,

$$\varepsilon = 1 - \frac{d}{d_0} \tag{S4}$$

where d is the experimental and d_0 is the theoretical density of the material.

The relative densities for the LSTO-H_2, LSTO-H_2-C and LSTO-H_2-in C samples are 94.0%, 96.0% and 89.3%, respectively. This means that d/d_0 ratios are equal to 0.94, 0.96 and 0.893 for three samples, respectively. By using equation S4, the ε of these samples can be obtained and then σ_0 is calculated according to equation S3. The obtained results at 330 K for the three LSTO samples prepared in this work are given in **Table S1**.

Supplementary figures

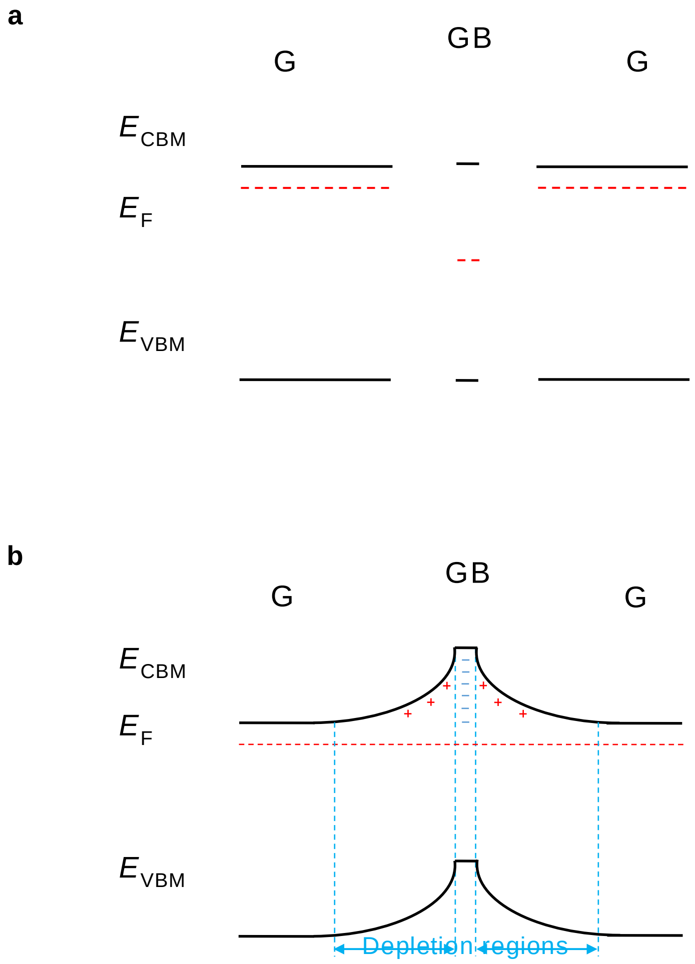

Figure S1. Energy band diagram of two $SrTiO_3$ grains and their boundary region, the grain boundary drawn with a finite length contains the grain boundary itself and the adjacent region in the grains where the oxygen vacancy becomes lower than the bulk grain. (a) Energy diagram of the two grains and their boundary region if they are isolated materials, the Fermi energy level of grain boundary region is deeper than the grains because of the depletion of donor, i.e., oxygen vacancy. (b) Energy diagram of the two grains and their boundary when they are brought into contact, the equilibrium of Fermi energy level causes the flow of electrons from grains to grain boundary region, which the raises of Fermi energy level in the boundary and bends the band upward in the grains.

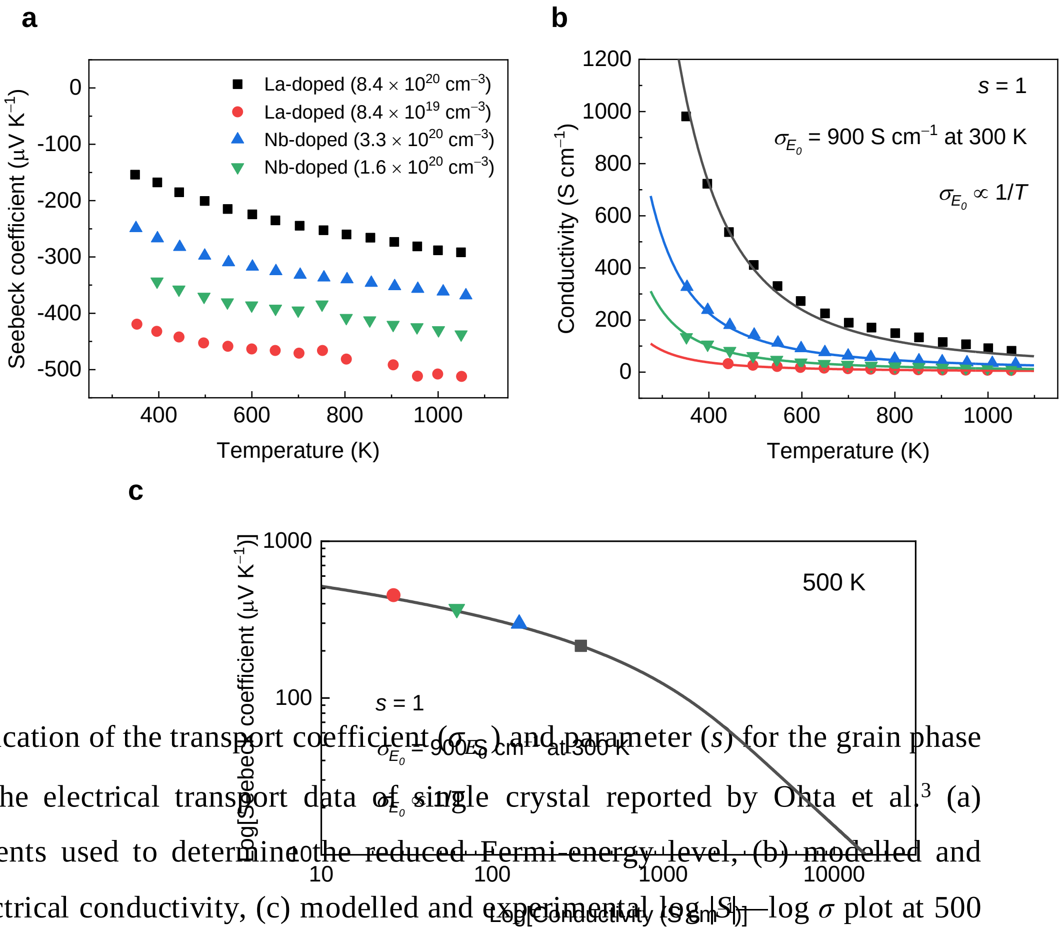

Figure S2. Verification of the transport coefficient (σ_{E_0}) and parameter (s) for the grain phase by fitting with the electrical transport data of single crystal reported by Ohta et al.[3] (a) Seebeck coefficients used to determine the reduced Fermi-energy level, (b) modelled and experimental electrical conductivity, (c) modelled and experimental log |S|—log σ plot at 500 K. The symbols are experimental data points and the solid lines are from the model. The modelled electrical conductivity and log |S|—log σ plot agreed well the trend for experimental values with σ_{E_0} = 900 S cm^{-1} and s = 1.

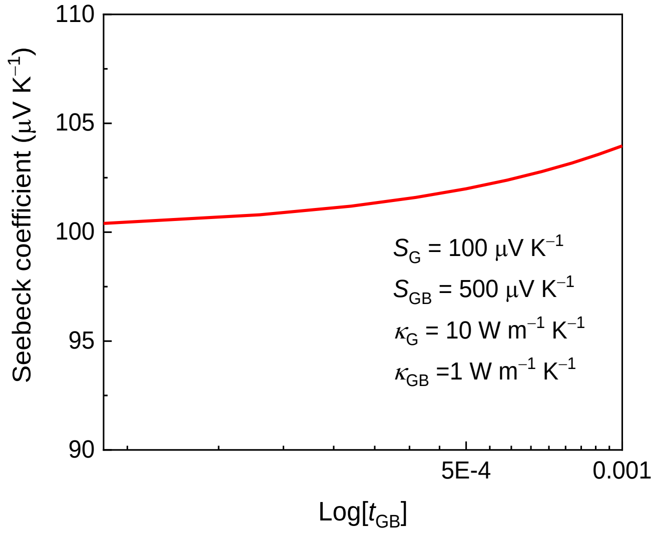

Figure S3. The plot of total Seebeck coefficients S as a function of t_{GB} calculated from equation S1 using parameters displayed in the figure, showing only slight variation of S (~ 2 μV K^{-1}) with the change of t_{GB} from 0.0005 to 0.001.

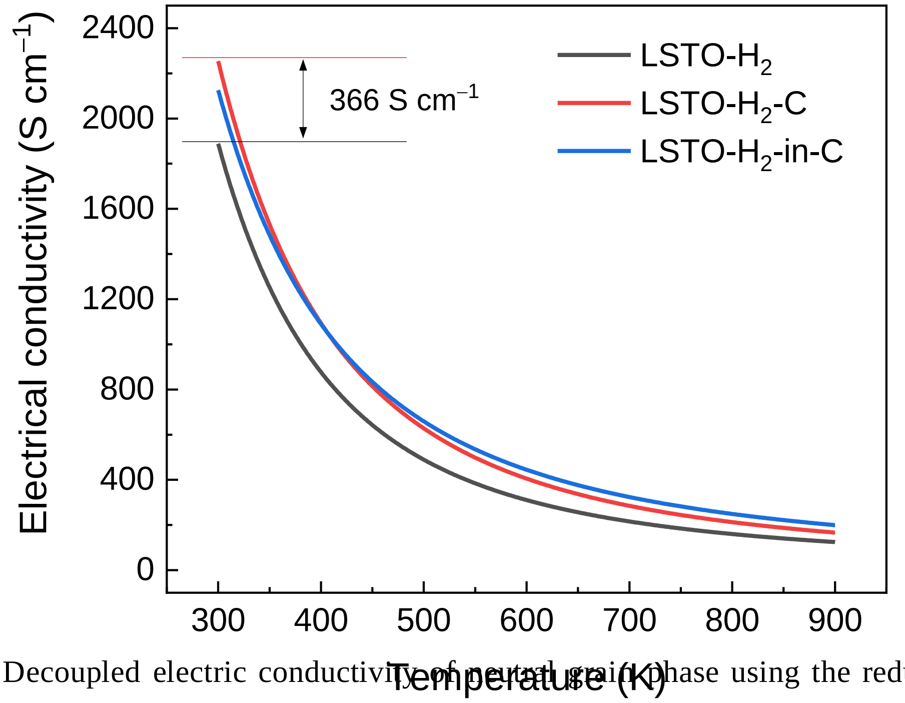

Figure S4. Decoupled electric conductivity of neutral grain phase using the reduced Fermi energy level extracted from the measured Seebeck coefficients, showing minor variation in the electrical conductivities of the neutral grain phases. For instance, the difference in the electrical conductivity values of the neutral grain phases for LSTO-H_2 and LSTO-H_2-in-C at 300 K is 366 S cm^{-1}, which is much smaller than that of the measured value from bulk ceramics (> 1000 S cm^{-1}, see Figure 6b).

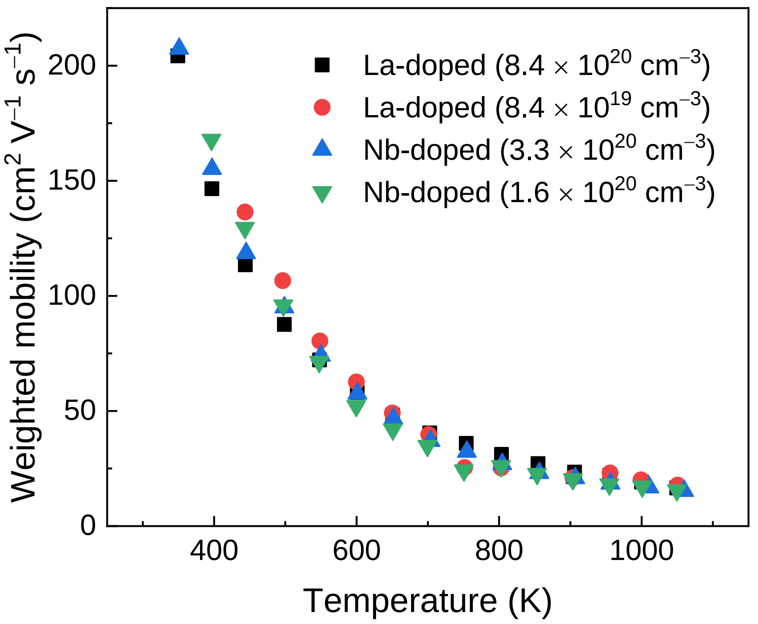

Figure S5. The computed weighted mobility of La- or Nb- doped single crystals with varying doping levels and thus carrier concentrations. Original transport data (Seebeck coefficient and electrical conductivity), for weighted mobility calculation, are from Ohta et al.[3]

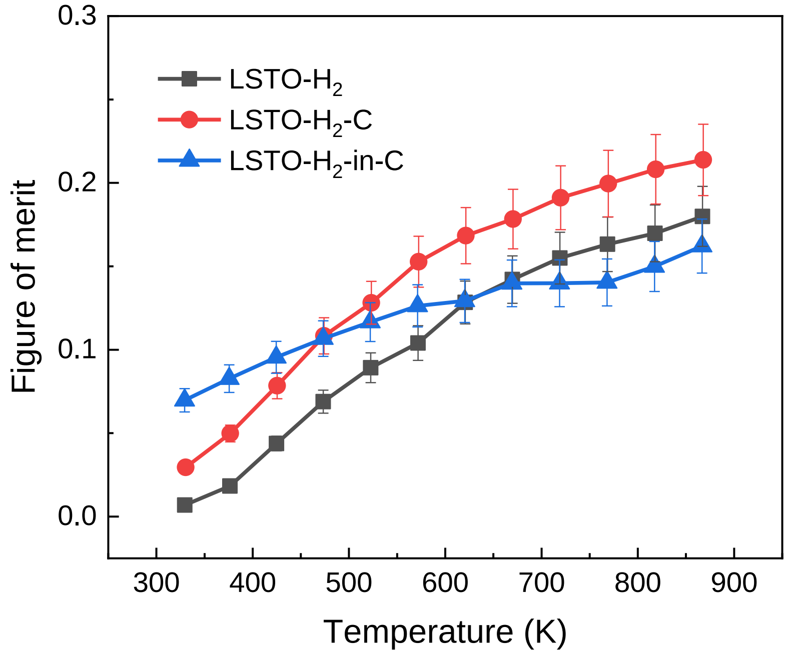

Figure S6. Dimensionless figure of merit *ZT* values for the three LSTO samples.

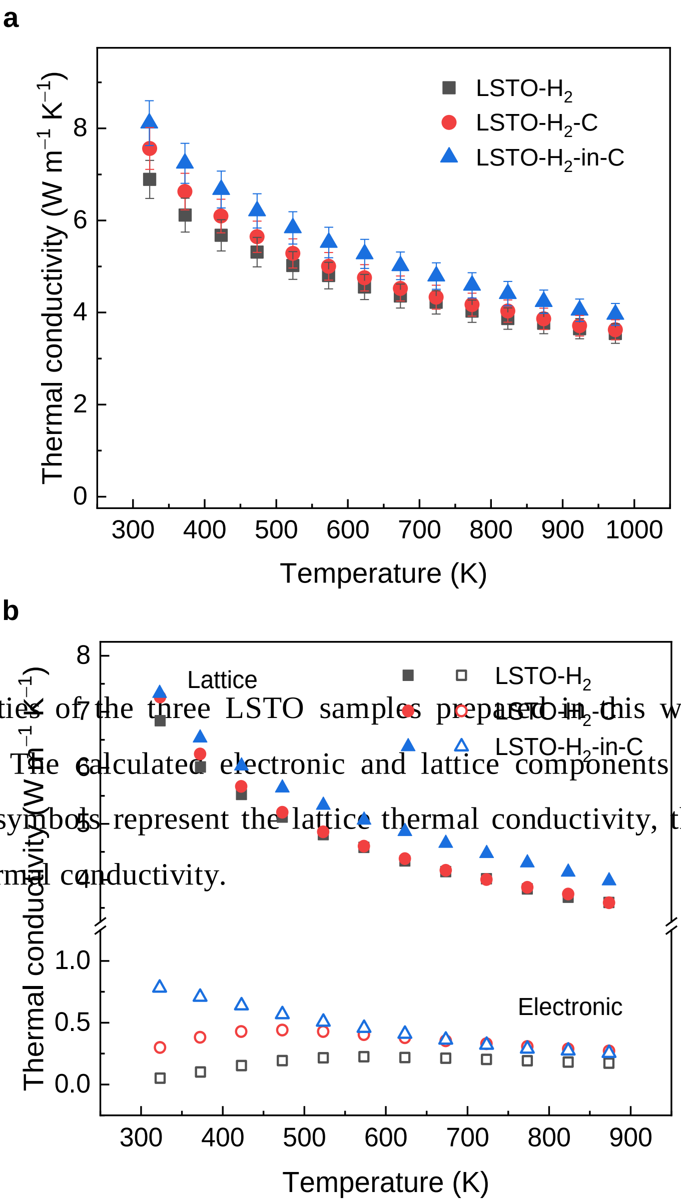

Figure S7. Thermal conductivities of the three LSTO samples prepared in this work. (a) Total thermal conductivity. (b) The calculated electronic and lattice components of total thermal conductivity; the solid symbols represent the lattice thermal conductivity, the open symbols represent electronic thermal conductivity.

Supplementary Tables

Table S1. Effects of porosity on the electrical conductivity. Ratio of experimental to theoretical density (d/d_0); porosity (ε); experimental conductivity (σ); effective conductivity (σ_0).

Sample	Relative density	d/d_0	ε	σ (S/cm)	σ_0 (S/cm)
LSTO-H_2	94.00%	0.940	0.060	85	93.14
LSTO-H_2-C	96.00%	0.960	0.040	492	522.75
LSTO-H_2-in C	89.30%	0.893	0.107	1288	1519.49

Table S2 Summary of published data for electrical conductivity (*σ*), Seebeck coefficients (*S*) and power factor (PF: $S^2\sigma$) for $SrTiO_3$-based materials at low temperatures (*T*).

Sample	*T* (K)	*σ* (S cm^{-1})	|*S*| (μV K^{-1})	PF ($S^2\sigma$) (μW m^{-1} K^{-2})	Ref.
La-$SrTiO_3$ single crystal	RT[a]	1000	150	2300	3
$La_{0.05}Sr_{0.95}Nb_{0.05}Ti_{0.95}O_3$	320	2000	50	600	4
$La_{0.067}Sr_{0.9}TiO_3$ + 0.6 wt% graphene	RT	2000	122	2500	5
$Sr_{0.95}Ti_{0.90}Ta_{0.10}O_3$	425	923	130	1351	6
$SrTiO_3$ + 0.64 vol% RGO	RT	16	310	150	7
$Sr_{0.95}Ti_{0.90}Nb_{0.10}O_3$	373	1260	110	1524	8
$SrTi_{0.9}Nb_{0.1}O_3$	RT	500	110	600	9
$Sr_{0.93}La_{0.07}Ti_{0.93}Nb_{0.07}O_3$	RT	900	90	700	10
$Sr_{0.9}La_{0.1}TiO_3$	330	390	123	590	11
$Sr_{0.98}Ti_{0.90}Nb_{0.10}O_3$ + 0.6 wt% RGO	330	2000	99	1980	12
$SrTiO_3$ + 0.7 wt.% RGO	RT	85	350	110	13
$Sr_{0.93}Ti_{0.9}Nb_{0.1}O_3$ + 0.6 wt.% RGO	RT	100	125	150	14
$La_{0.080}Sr_{0.9}TiO_3$	RT	1423	111	1745	This work

[a]Room temperature (RT)

References:

(1) Kuo, J. J.; Kang, S. D.; Imasato, K.; Tamaki, H.; Ohno, S.; Kanno, T.; Snyder, G. J. Grain Boundary Dominated Charge Transport in Mg_3Sb_2-Based Compounds. *Energy Environ. Sci.* **2018,** *11*, 429-434.

(2) Zhang, L.; Tosho, T.; Okinaka, N.; Akiyama, T. Thermoelectric Properties of Solution Combustion Synthesized Al-Doped ZnO. *Mater. Trans.* **2008,** *49*, 2868-2874.

(3) Ohta, S.; Nomura, T.; Ohta, H.; Koumoto, K. High-Temperature Carrier Transport and Thermoelectric Properties of Heavily La- or Nb-Doped $SrTiO_3$ Single Crystals. *J. Appl. Phys.* **2005,** *97*, 034106.

(4) Teranishi, T.; Ishikawa, Y.; Hayashi, H.; Kishimoto, A.; Katayama, M.; Inada, Y. Thermoelectric Efficiency of Reduced $SrTiO_3$ Ceramics Modified with La and Nb. *J. Am. Ceram. Soc.* **2013,** *96*, 2852-2856.
(5) Lin, Y.; Norman, C.; Srivastava, D.; Azough, F.; Wang, L.; Robbins, M.; Simpson, K.; Freer, R.; Kinloch, I. A. Thermoelectric Power Generation from Lanthanum Strontium Titanium Oxide at Room Temperature through the Addition of Graphene. *ACS Appl. Mater. Interfaces* **2015,** *7*, 15898-908.
(6) Yaremchenko, A. A.; Populoh, S.; Patrício, S. G.; Macías, J.; Thiel, P.; Fagg, D. P.; Weidenkaff, A.; Frade, J. R.; Kovalevsky, A. V. Boosting Thermoelectric Performance by Controlled Defect Chemistry Engineering in Ta-Substituted Strontium Titanate. *Chem. Mater.* **2015,** *27*, 4995-5006.
(7) Feng, X.; Fan, Y.; Nomura, N.; Kikuchi, K.; Wang, L.; Jiang, W.; Kawasaki, A. Graphene Promoted Oxygen Vacancies in Perovskite for Enhanced Thermoelectric Properties. *Carbon* **2017,** *112*, 169-176.
(8) Kovalevsky, A. V.; Aguirre, M. H.; Populoh, S.; Patrício, S. G.; Ferreira, N. M.; Mikhalev, S. M.; Fagg, D. P.; Weidenkaff, A.; Frade, J. R. Designing Strontium Titanate-Based Thermoelectrics: Insight into Defect Chemistry Mechanisms. *J. Mater. Chem. A* **2017,** *5*, 3909-3922.
(9) Wang, J.; Zhang, B. Y.; Kang, H. J.; Li, Y.; Yaer, X.; Li, J. F.; Tan, Q.; Zhang, S.; Fan, G. H.; Liu, C. Y.; Miao, L.; Nan, D.; Wang, T. M.; Zhao, L. D. Record High Thermoelectric Performance in Bulk $SrTiO_3$ Via Nano-Scale Modulation Doping. *Nano Energy* **2017,** *35*, 387-395.
(10) Li, J. B.; Wang, J.; Li, J. F.; Li, Y.; Yang, H.; Yu, H. Y.; Ma, X. B.; Yaer, X.; Liu, L.; Miao, L. Broadening the Temperature Range for High Thermoelectric Performance of Bulk Polycrystalline Strontium Titanate by Controlling the Electronic Transport Properties. *J. Mater. Chem. C* **2018,** *6*, 7594-7603.
(11) Azough, F.; Gholinia, A.; Alvarez-Ruiz, D. T.; Duran, E.; Kepaptsoglou, D. M.; Eggeman, A. S.; Ramasse, Q. M.; Freer, R. Self-Nanostructuring in $SrTiO_3$: A Novel Strategy for Enhancement of Thermoelectric Response in Oxides. *ACS Appl. Mater. Interfaces* **2019,** *11*, 32833-32843.
(12) Okhay, O.; Zlotnik, S.; Xie, W.; Orlinski, K.; Hortiguela Gallo, M. J.; Otero-Irurueta, G.; Fernandes, A. J. S.; Pawlak, D. A.; Weidenkaff, A.; Tkach, A. Thermoelectric Performance of Nb-Doped $SrTiO_3$ Enhanced by Reduced Graphene Oxide and Sr Deficiency Cooperation. *Carbon* **2019,** *143*, 215-222.
(13) Rahman, J. U.; Du, N. V.; Nam, W. H.; Shin, W. H.; Lee, K. H.; Seo, W. S.; Kim, M. H.; Lee, S. Grain Boundary Interfaces Controlled by Reduced Graphene Oxide in Nonstoichiometric $SrTiO_{3-\delta}$ Thermoelectrics. *Sci. Rep.* **2019,** *9*, 8624.
(14) Wu, C.; Li, J.; Fan, Y.; Xing, J.; Gu, H.; Zhou, Z.; Lu, X.; Zhang, Q.; Wang, L.; Jiang, W. The Effect of Reduced Graphene Oxide on Microstructure and Thermoelectric Properties of Nb-Doped a-Site-Deficient $SrTiO_3$ Ceramics. *J. Alloys Compd.* **2019,** *786*, 884-893.
